# Worms need microbes too: microbiota, health and aging in *Caenorhabditis elegans*

**DOI:** 10.1002/emmm.201100972

**Published:** 2013-08-01

**Authors:** Filipe Cabreiro, David Gems

**Affiliations:** Institute of Healthy Ageing, and Research Department of Genetics, Evolution and Environment, University College LondonLondon, UK

**Keywords:** aging, *C. elegans*, metformin, microbiota, type-2 diabetes

## Abstract

Many animal species live in close association with commensal and symbiotic microbes (microbiota). Recent studies have revealed that the status of gastrointestinal tract microbiota can influence nutrition-related syndromes such as obesity and type-2 diabetes, and perhaps aging. These morbidities have a profound impact in terms of individual suffering, and are an increasing economic burden to modern societies. Several theories have been proposed for the influence of microbiota on host metabolism, but these largely remain to be proven. In this article we discuss how microbiota may be manipulated (via pharmacology, diet, or gene manipulation) in order to alter metabolism, immunity, health and aging in the host. The nematode *Caenorhabditis elegans* in combination with one microbial species is an excellent, defined model system to investigate the mechanisms of host–microbiota interactions, particularly given the combined power of worm and microbial genetics. We also discuss the multifaceted nature of the worm–microbe relationship, which likely encompasses predation, commensalism, pathogenicity and necromeny.

## Introduction

Animals and plants rarely live in isolation but rather exist in intimate association with other species — particularly microorganisms. The nature of host–microbe interactions is defined according to which participant experiences benefit or harm (Rosenberg & Zilber-Rosenberg, 2011). For example, host benefit with harm to the microbe occurs in a host–predator relationship, while host harm with microbe benefit occurs in host–pathogen relationships. More characteristic of the symbiotic relationship between host and intestinal microbiota is commensalism, where there is benefit to only one partner of this interaction, and mutualism, where there is benefit to both.

In mammals, host–microbe symbiotic interactions mainly occur along mucosal surfaces, with the most important one being the intestinal mucosa. The intestine harbours a vast population of symbiotic microbes that outnumber host somatic cells by 10-fold and are capable of performing varied metabolic and protective functions. Along the intestinal tract, bi-directional host–microbiota exchanges occur with mutual benefits to both organisms. Indeed, 10% of the metabolites in the mammalian blood flow are of bacterial origin (Wikoff et al, 2009). For instance, bacteria in the gut are capable of synthesizing and excreting vitamins, which can be absorbed by the host. The gut microbiota also plays an important role in catabolizing dietary fibre for human nutrition (Nicholson et al, 2012). The microbiota — referred to as “the forgotten organ” (O'Hara & Shanahan, 2006) — is thus involved in many metabolic processes with important consequences to host physiology and in the modulation of metabolic phenotypes. Consistent with this, sequence analysis shows that the microbiome is importantly enriched in genes encoding metabolic activities such as metabolism of xenobiotic compounds, amino acids and carbohydrates (Greiner & Backhed, 2011). Importantly, the microbiota contributes to the fitness of the host by influencing development (Rawls et al, 2004), reproduction (Shimizu et al, 1998), metabolism (Cani & Delzenne, 2009; Nicholson et al, 2012), immunity (Kau et al, 2011) and lifespan (Ottaviani et al, 2011).

Gut microbiota status is also a determinant of health in the host. Consequently, human health may be affected by factors that alter microbiota, *e.g*. dietary changes and antibiotic usage, which may reduce microbiota complexity (Maurice et al, 2013; Muegge et al, 2011). This has led to concern that disruption of microbiota could be contributing to conditions such as obesity, diabetes, metabolic syndrome, autoimmune disorders, inflammatory bowel disease, cancer and aging (Cho & Blaser, 2012; Claesson et al, 2012). Thus, it is important to try to understand how these microbial communities determine human health status, including healthy aging.

## *C. elegans* as a model for host–microbiota interactions

The suspected role of host–microbiota interactions in human disease is largely derived from observational studies, and it is often difficult to establish whether changes in microbiota are cause or effect of their associated pathologies. The use of mouse models offers one solution, but the complexity of mouse microbiota presents a challenge, and mouse costs are another impediment (Claesson et al, 2012).

*C. elegans* has proven to be an excellent model for the study of many central processes in biology, including energy metabolism (Jones & Ashrafi, 2009), immunity (Schulenburg et al, 2004) and aging (Kenyon, 2010). Could the worm also serve as a model to understand how microbiota affect host physiology in the context of disease and aging? Several features of *C. elegans* make it well-equipped for such a role. *C. elegans* are easily maintained monoxenically (*i.e*. with one bacterial species) in laboratory conditions. Growth and maintenance of the bacterial food source is achieved through standard microbiology technique. Nematode growth medium agar, which has been aseptically poured into Petri plates supports growth of seeded bacteria which in turn supports *C. elegans* growth and reproduction (Brenner, 1974). In terms of its general suitability as a model organism, these include the transparency of its body wall, which allow the internal organs, fat depots and age-related pathology to be viewed; a very short lifespan (2–3 weeks); and excellent genetics, including a sequenced and fully annotated genome, and availability of mutants and reagents for gene knock out. Moreover, fluorescently tagged bacteria may be readily visualized within the transparent body of *C. elegans* providing easy assessment of gut colonization (Sifri et al, 2005).

In terms of suitability for host–microbiota studies, the intestine is the largest somatic organ in the worm, and it is typically full of microbes (Felix & Duveau, 2012; Garigan et al, 2002; McGee et al, 2011). The *C. elegans* intestine is a non-renewable 20-cell monolayer arranged to form a tube with a central lumen (Brenner, 1974). The intestinal cells have a brush border on their apical side, comprising finger-like microvilli attached to a terminal web of actin and intermediate filaments (Troemel et al, 2008). Functionally, and as in humans, the intestine constitutes a first line of defence against a potential harmful environment (*e.g*. pathogens; Ewbank & Zugasti, 2011; Lievin-Le Moal & Servin, 2006) but at the same time provide a means for nutrient extraction, storage and absorption, as well as waste excretion. In addition, the intestine plays a major role in organismal health and lifespan, and acts as a signalling centre influencing other tissues and modulated by bacterially derived cues (Rera et al, 2013).

**Glossary**

**Biotransformation**

Chemical alteration by an organism of a chemical compound. These can include nutrients, toxins, xenobiotics.

**Commensalism**

From the medieval Latin *com mensa* or ‘sharing the same table’. Relationship between individuals of two species, in which one species obtains benefits from the other without either harming or benefiting the latter.

**Dietary restriction**

Reduction of dietary intake without starvation, which reduces fertility and increases lifespan in many animal species. Also known as caloric restriction.

**Dysbiosis**

State of microbial imbalance in the gut leading to host dyshomeostasis.

**Gut microbiota**

The collective community of microbes inhabiting a host gastrointestinal tract. In contrast to worms in the wild, nematodes in the laboratory are usually raised in the presence of a single bacterial strain.

**Holobiont**

An organism and its associated symbiotic microbes. This is the unit of selection in the hologenome theory.

**Microbiome**

The collective genomic content of an individual's microbiota.

**Mutualism**

A relationship between individuals of different species in which both species benefit from the interaction.

**Necromeny**

The behaviour of an organism that attaches itself to a host organism, and then feeds upon its decomposing cadaver after death.

**Pathobiont**

Member species of the microbiota that under conditions of host and/or microbiota dyshomeostasis can cause pathology. Distinct from pathogen.

**Probiotic**

Live organisms that when ingested confer health benefits to the host either by interacting directly with the host or through modulation of other members of the microbiota. Bacterial strains from the genera *Lactobacillus* and *Bifidobacterium* are candidate probiotics.

**Symbiont**

An organism in a mutually beneficial relationship with another.

**Symbiosis**

Close and long-term beneficial interaction between two species.

**Type-2 diabetes**

Pandemic disorder characterized by high blood glucose in the context of relative insulin deficiency and insulin resistance. About 6% of the world population suffers from this metabolic disorder.

**Worm-bug**

Term coined here as an extension of the holobiont concept. Refers to the nematode–microbe pair as an indivisible entity in terms of viability and evolution.

The use of a model of a host containing single microbial species as its microbiota has major advantages in terms of experimental tractability, particularly where both species are well-developed model organism with extensive genetics.

## Worms and bacteria: a marriage made by evolution

Gut microbiota has evolved with humans as a mutualistic partner. The hologenome theory proposes that the host and its symbiotic microbiota (holobiont) is a unit of selection in evolution (Rosenberg & Zilber-Rosenberg, 2011). The hologenome is the extended genome resulting from the combination of the host genome and that of the microbiota (microbiome). In the human gut, 10^14^ bacterial cells (Savage, 1977) provide the host with more than 4,500,000 genes (Collison et al, 2012). Thus, the microbiota and its microbiome offer genetic and metabolic features, allowing us to evolve from within and increasing our fitness when challenged by new environments.

Variation is required for evolution and increased fitness. Some of the mechanisms for introducing variation into holobionts are the acquisition of new symbionts from the environment or through horizontal gene transfer between different bacterial species. In fact, horizontal gene transfer has been observed within the *C. elegans* gut and is modulated by host aging and genotype (Portal-Celhay et al, 2013). Moreover, aging in the worm facilitates intraluminal genetic transfer due to increased persistence of founding colonizing bacterial strains. Thus, selection continuously occurs and dictates interspecies and intrastrain competition for survival (Portal-Celhay & Blaser, 2012). Also, consistent with co-evolution of bacterial and worm species, it was recently observed that two bacterial non-coding RNAs alter the behaviour and lifespan of the *C. elegans* host by altering gene expression (Liu et al, 2012). These observations are consistent with the occurrence of interaction between the worm and bacterial genome, consistent with the hologenome theory, and the view that worm and microbiota represent a single unit of selection in the co-evolution of both partners.

The evolution of *C. elegans* — at very least, its emergence from its terrestrial ancestor species — must have occurred in the constant presence of intestinal microbes. It is therefore probable that aspects of biological function in the worm have evolved to operate in conjunction with and assisted by microbial functions. This suggests that it may only be possible to fully understand worm metabolism as part of a worm–microbe holobiont. For convenience, we suggest the briefer term *worm]bug* to refer to the single, functional entity that is the worm–microbe holobiont.

## Fitness in the worm-bug: bacteria as more than just a food source

When freshly isolated from the wild, *C. elegans* and other terrestrial nematodes often harbour a diverse bacterial flora in their gut lumen, reminiscent of the microbial communities of higher organisms (Bumbarger et al, 2013; Felix & Duveau, 2012). By contrast, in the laboratory *C. elegans* is typically maintained in monoxenic culture, *i.e*. in the presence of single bacterial strain (Brenner, 1974). Most often, this is the gram-negative bacterium *Escherichia coli*, native to the human bowel, but other species are sometimes used, such as the gram-positive *Bacillus subtilis* (Garsin et al, 2003).

**Figure 1:**
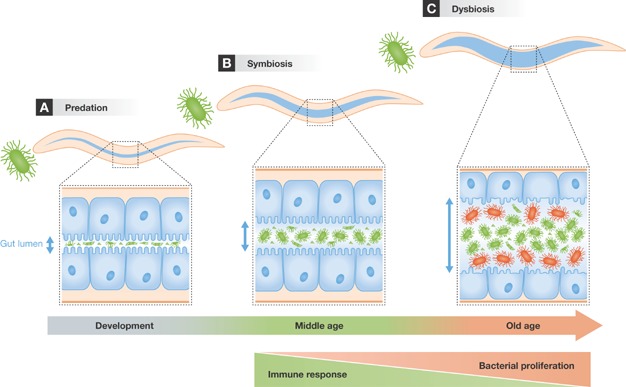
From hunter to prey: the changing relationship between *C. elegans* and *E. coli* Different stages in the course of the life of the worm can serve as models to study how humans interact with their microbiota. During development, bacteria mainly serve as a source of food (green), since they are almost entirely crushed by the pharyngeal grinder and live colonies are absent from the lumen of the gut (Kurz et al, 2003).In young adults, bacteria that have escaped the action of the grinder proliferate and establish a community within some sections of the lumen of the gut (Portal-Celhay et al, 2012), and exist as commensals or symbionts (green).As the worm ages, bacteria proliferating within the lumen of the gut become detrimental to the host (McGee et al, 2011) and can cause severe constipation (Garigan et al, 2002). A possibility is that this reflects changes in bacterial metabolism engendering dysbiosis (red). In addition, blockage of the lumen (Garigan et al, 2002), and changes in bacterial metabolism may be detrimental too (Portal-Celhay et al, 2012). Treatments that block bacterial proliferation extend lifespan, likely by preventing microbial dysbiosis (Garigan et al, 2002; Gems & Riddle, 2000).

The *E. coli* with which *C. elegans* is cultured was initially viewed merely as a food source for the worm, and more recently as mildly pathogenic (Garigan et al, 2002; Gems & Riddle, 2000). The relationship between worm and microbe changes during the course of life: initially the former is the predator and the latter the prey, but later in life this relationship shifts into reverse. Maintenance of the predator role of the worm is dependent upon the effectiveness of the pharyngeal grinder to disrupt bacteria (Portal-Celhay et al, 2012), bacterial clearance by digestion (Kim & Mylonakis, 2012), host defences (Pukkila-Worley & Ausubel, 2012), defecation (Rae et al, 2012) and the proliferative capacity of the bacterial strain (Portal-Celhay & Blaser, 2012; Fig 1). An adult worm contains approximately 10,000 bacterial cells, a number 10-times greater than that of host worm somatic cells (Portal-Celhay et al, 2012): perhaps coincidentally, this microbiota-to-host cell ratio is similar to that found in humans (Savage, 1977).

What happens to *E. coli* when they are consumed by *C. elegans* remains poorly understood, particularly the process of digestion in the intestinal lumen (Walker et al, 2005). The nematode *Pristionchus pacificus*, unlike *C. elegans*, has no pharyngeal grinder for masticating bacteria (Bumbarger et al, 2013) and is seemingly able to subsist on intact *E. coli* in its intestine. So, how do bacteria nourish worms? In *C. elegans*, the provision of different *E. coli* strains as food sources can profoundly affect the worm transcriptome (Macneil et al, 2013), metabolome (Reinke et al, 2010), intestinal fat storage (Brooks et al, 2009) and lifespan (Brooks et al, 2009; Macneil et al, 2013; Reinke et al, 2010). Yet fat storage levels in worms are not a simple reflection of the carbohydrate or fatty acid composition of the bacterial strain consumed (Brooks et al, 2009). This study suggests that other nutritional cues perceived in the intestine could regulate host metabolism. In mammals, gut microbiota plays an important role in providing essential metabolites such as folate and other vitamins. Similarly, alterations of bacterial folate metabolism modulate *C. elegans* lifespan (Virk et al, 2012).

Critically, worms have a nutritional requirement for metabolically active bacteria: if provided with a nutrient-rich but microbe-free diet, their development is severely retarded, fertility greatly reduced and lifespan increased (probably due to dietary restriction (DR)) (Houthoofd et al, 2002). Normal growth, fertility and aging is restored by addition of live *E. coli*, and add-back experiments with live *versus* dead *E. coli* imply that the critical “dietary” component provided by *E. coli* is their metabolic activity (Lenaerts et al, 2008). Thus, *C. elegans* appears to require microbiota for normal growth and metabolism. Supporting this view, worm lifespan can be increased by mutational alteration of metabolism in *E. coli* affecting respiration (Saiki et al, 2008).

Like *E. coli*, *B. subtilis* also modulates the physiology of its worm host independently of its role as food (Laaberki & Dworkin, 2008). Worms grown on dividing vegetative *B. subtilis* cells are longer-lived than worms grown in the presence of *E. coli* (Garsin et al, 2003). Recently, it has been shown that *B. subtilis* extends lifespan of the worm host by production of nitric oxide inside the gut (Gusarov et al, 2013).

Again, in mammals early life interactions between host and pathogen are critical to the maturation of the host immune system (von Mutius et al, 2000). Similarly, in nematodes, exposure during development to more pathogenic forms of *E. coli* can increase resistance to the same or other pathogens in the adult, and also to other stressors (Leroy et al, 2012).

**Figure 2:**
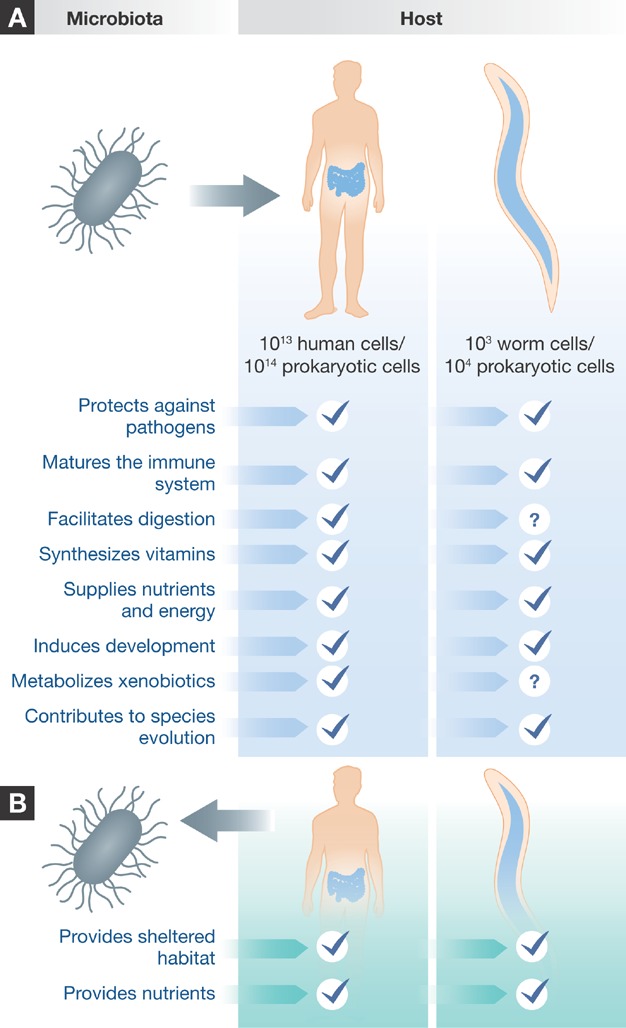
Functions of the microbiota in human and nematode hosts Schematic summary of the effects of intestinal microbiota on host fitness. These include protective, structural and metabolic effects.The host confers benefits to the microbiota. In the case of *C. elegans*, the benefits to the microbiota, as hypothesized here, involve cannibalistic commensalism and necromeny.

Overall, these observations support the view that bacteria can modulate worm physiology and provide nutritional requirements independently of its role as a food source, *i.e*. that it functions as microbiota for *C. elegans* (Fig 2).

### The role of probiotics in worm physiology

A century ago, Élie Metchnikoff speculated that gut bacteria contributes to aging, and that consumption of fermented milk (yoghurt) could protect against such harmful bacterial effects (Metchnikoff, 1910). While the idea that yoghurt is an elixir is entirely doubtful, research in the last decades has provided some support for the view that probiotic bacteria (*i.e*. bacteria in the diet that alter the composition of the microbiota in a beneficial way) can exert benign effects, including immunomodulation, antimicrobial effects and nutritional supplementation (Rauch & Lynch, 2012). However, the mechanisms underlying possible beneficial effects of probiotics remain poorly understood. In *C. elegans*, both live (Ikeda et al, 2007; Kim & Mylonakis, 2012; Wang et al, 2011) and dead (Lee et al, 2011) probiotic bacteria (*i.e. Lactobacillus* and *Bifidobacterium*) can enhance immune defences and increase lifespan (Komura et al, 2013). Thus, *C. elegans* could be of use for investigating dietary manipulation with probiotics.

### Benefits to the microbe in the worm-bug?

In mammals, the host–microbiota relationship is fully symbiotic, at least for some microbes insofar as they benefit from the interaction, acquiring from their hosts a sheltered and nutrient-rich environment (Fraune & Bosch, 2010). Growth promoting secretions by the epithelia (*e.g*. nutrients, antimicrobials) act as a selective force within the gut. This feature of the host provides competitive advantages of certain slow growing beneficial bacterial strains that would otherwise be lost (Schluter & Foster, 2012). Similarly, the intestinal environment of the worm constitutes an *in vivo* selection medium for better-adapted commensal colonizers within an evolving bacterial population. This adaptation allows commensals to compete more efficiently against pathogenic strains for gut colonization (Portal-Celhay & Blaser, 2012). Furthermore, the intestinal environment, possibly providing nutrients, also allows the germination and consequent digestion of *B. subtilis* spores to support worm growth (Laaberki & Dworkin, 2008).

Such mutualistic interactions illustrate how symbionts can benefit from and provide advantage to the host. Yet how *E. coli* might benefit from its interaction with *C. elegans* remains unclear. One scenario is cannibalistic commensalism: *E. coli* in the gut lumen eat at the same table as their host, sharing the meal that is their masticated siblings. Another possibility is biological dispersal of the symbiont to bacterial-nutrient rich environments to support growth of the worm-bug (Lee et al, 2012). A third is necromeny: *i.e*. the microbes that survive their early role as prey reap the benefit of a meal from the corpse of their host.

## Bacterial dysbiosis as a cause of pathology in the worm?

In mammals, dysbiosis in the microbiota is characterized by an imbalance of microbial communities. Diseases associated with dysbiosis include obesity, inflammatory bowel disease (IBD) and diabetes (Clemente et al, 2012). In obese or type-2 diabetic animals, changes in the composition of the microbiota are associated with increased permeability of the intestine and leakage of gram-negative-associated lipopolysaccharide to promote low grade inflammation that characterize these disease states (Cani & Delzenne, 2009). *C. elegans* can be used as a model to study the pathophysiology of intestinal inflammation (*e.g*. inflammatory bowel disease and necrotic enterocolitis; Lin & Hackam, 2011) and other diseases associated with metabolic syndrome, such as non-alcoholic fatty liver disease (Walker et al, 2011).

However, it remains unknown whether something akin to dysbiosis exists in *C. elegans*, particularly those in monoxenic culture. One possibility is that in worms in monoxenic culture dysbiosis involves physiological changes in the bacteria caused by extrinsic factors (*e.g*. environmental stressors, growth conditions) or intrinsic factors (*e.g*. aging, either of the host or the bacterial population in the gut lumen). During worm aging, the intestine shows symptoms of pathology, including increased variability in shape and size of the intestinal lumen, loss or shortening of microvilli and loss of intestinal nuclei (McGee et al, 2011). In addition, the intestinal lumen is frequently distended and packed with undigested bacteria (Garigan et al, 2002; McGee et al, 2011) that can become detrimental to microvillar structures (Fig 1). Thus, it is possible that in adverse conditions *E. coli* in the gut lumen alter their physiology and cause pathology (*i.e*. become pathobionts). Since *E. coli* have not been observed to invade intestinal cells it is conceivable that molecules secreted by pathobiont *E. coli* are responsible for the detrimental effects on the host. In fact, bacterial lipopolysaccharides have a regulatory role in the lifespan of the worm (Maier et al, 2010). Recently it has been shown that restoration of the O antigen in *wbbl* deficient *E. coli* K-12 increases bacterial colonisation and shortens nematode lifespan (Browning et al, 2013). Whether these molecules can exert an additional physiological role in old worms remains unexplored.

### Is intestinal colonization deleterious *per se*?

Another form that dysbiosis might take in worms is microbial over-proliferation in the gut lumen. *C. elegans* possess innate immunity, which is regulated by insulin/IGF-1, p38 MAP kinase and TGF-b signalling (Pukkila-Worley & Ausubel, 2012). Gut bacterial load is dependent on bacterial proliferative capacity (Gomez et al, 2012) and regulated by host innate immunity (Portal-Celhay et al, 2012). As worms age, there is a decline in innate immunity (Youngman et al, 2011) and in other intestinal functions, including ingestion and defecation (Smith et al, 2008); consequently, old worms are less able to keep their microbes at bay and in low numbers. An observed inverse correlation between bacterial counts with lifespan (Gomez et al, 2012; Portal-Celhay et al, 2012) and mortality rate (Baeriswyl et al, 2010) suggests that colonization contributes to aging. However, pharyngeal grinder defective mutants in the presence of non-pathogenic strains show higher bacterial load and are short-lived only if their innate immune system is inefficient (Portal-Celhay et al, 2012). In addition, other bacterial strains such as *Enteroccocus faecium* colonize the worm gut heavily without causing significant mortality (Garsin et al, 2001). Thus, colonization with non-pathogenic strains may only increase mortality when the immune system deteriorates with age.

As in the worm, peristalsis in rodents acts as a first line of defence against bacterial colonization by preventing the adherence of bacteria to intestinal cells. Defects in peristalsis (*e.g*. constipation) lead to increased intestinal transit time, changes in microbiota composition, intestinal permeability and systemic immune responses (Khalif et al, 2005). In flies, aging of the intestine has a significant impact on the lifespan of the animal (Rera et al, 2013). In nematodes, defects in defecation can reduce survival in the presence of pathogens (Rae et al, 2012). In addition, treatment of the bacteria that reduces worm constipation can increase lifespan (Garigan et al, 2002; Gems & Riddle, 2000). Overall, evidence suggests that maintaining a healthy, functional gut during aging is an important determinant of lifespan and that intestinal colonisation is a key contributory factor.

### Is dietary restriction a function of the microbiota?

Besides reducing fertility and extending lifespan, DR, the controlled reduction of food intake without starvation, can reduce incidence of many age-related diseases, including obesity, type-2 diabetes and many forms of cancer (Masoro, 2005). DR can also alter the microbiota. For example in humans, studies with participants of the Calorie Restriction Society, who keep accurate records of their diets, show that within an individual the changes occurring in the structure (relative composition of the different microbes) and function (metabolic activities and associated end-products) of the gut microbiome are associated with dietary fibre intake and total protein intake, respectively (Muegge et al, 2011). Indeed, several dietary constituents (*e.g*. non-digestible carbohydrates) have an important impact on gut microbiota composition, with specific bacterial groups modulated by specific dietary interventions (Scott et al, 2011). This raises the possibility that alteration in microbiota contribute to DR's beneficial effects on health. Supporting this idea, both the absence of microbiota and DR extend rodent lifespan but the combination of both had no further additive effects (Snyder et al, 1990; Tazume et al, 1991). Similarly, absence of microbiota in adult *C. elegans* extends lifespan (Houthoofd et al, 2002; Kaeberlein et al, 2006). In dogs, microbiota-derived metabolic markers are altered both during aging and by a 25% reduction in food intake (Wang et al, 2007). In flies, acetic acid production by a commensal bacterium modulates insulin/insulin-like growth factor signalling (IIS) to regulate host development and metabolic homeostasis (Shin et al, 2011). Possibly, the microbiota exerts a signalling role through its metabolites to mediate a variety of systemic responses of DR.

Various methods have been used to subject *C. elegans* to DR, including bacterial dilution, use of feeding defective mutant worm strains and axenic (microbe-free) culture (Greer & Brunet, 2009; Mair et al, 2009). Longevity induced by different modes of DR can be dependent on different longevity control genes (Greer & Brunet, 2009), implying that DR in worms can act by more than one mechanism (Mair et al, 2009).

In the marriage of worm and microbe, the latter plays three roles: those of food, commensal and pathogen. This defines at least three mechanisms by which reducing *E. coli* availability could increase worm lifespan: reducing nutrient availability, reducing microbial metabolic functions essential for *C. elegans* and reducing pathogenicity. Moreover, different DR protocols may differentially affect metabolic status in the bacteria (Table 1). Again, the *C. elegans*–microbe dual model organism system provides an excellent and tractable model for investigating the role of host–microbiota interactions as potential mediators of the effects of DR.

**Table 1:** Dietary restriction methods used in *C. elegans*

Method	Medium	Bacterial treatment	Bacterial state	% of mean lifespan extension	Refs.
bDR	Liquid	Antibiotics	Non-proliferating	29–57	Klass (1977)
DP	Solid	Peptone reduction	Non-proliferating	33	Hosono et al (1989)
*eat-2*	Solid	None	Proliferating	57	Lakowski & Hekimi (1998)
Axenic	Liquid/solid	NA	NA	85/74	Houthoofd et al (2002), Lenaerts et al (2008)
DD	Solid	NA	NA	61	Kaeberlein et al (2006)
sDR	Solid	None/UV-irradation	Proliferating/quiescent	35/18	Greer et al (2007)
IDR	Liquid	Antibiotics	Non-proliferating	28	Bishop & Guarente (2007)

The standard *C. elegans* laboratory culture condition is in a lawn of live bacteria (*E. coli* OP50) on an agar plate containing nematode growth medium. The DR methods in worms are: diluting the bacteria in liquid medium: bacterial DR (bDR); restriction of bacterial growth by the dilution of peptone in nutrient agar (DP); use of mutations (usually in the gene *eat-2*) that reduce the pharyngeal pumping rate of the worms; use of semi-defined axenic medium (axenic); the total absence of bacteria on plates (DD); serially diluted DR (sDR); and shallow liquid culture on agar plates; liquid DR (lDR). N.A., not applicable. Clearly, some of these protocols are very similar, *e.g*. DD and solid axenic culture, and bDR and sDR. DR research in *C. elegans* would presumably benefit from rationalization and standardization of DR methodologies.

## Microbiota as a mediator of drug effects on health and aging

Microbiota can also play a role in drug action, either through effects on their availability (*e.g*. via biotransformation) or because drug effects on microbiota affect the host. In terms of biotransformation: to understand action of a given drug, it is useful to know if such biotransformation occurs and, if so, which microbial species effect this (Haiser & Turnbaugh, 2012). For instance, the efficacy of many widely used drugs, including statins, is likely determined by both the microbiota and host genetic background (Kaddurah-Daouk et al, 2011). Sulfasalazine, a drug to treat rheumatoid arthritis in humans, undergoes azo reduction by the microbiota, and also induces expression changes in bacterial genes encoding thioredoxins and nitrate reductases (Maurice et al, 2013).

In terms of microbiota as mediators of drug effects on the host: some drugs certainly do affect microbiota. Antibiotics are usually prescribed to facilitate clearance of bacterial infection. However, they can strongly perturb commensal microbial communities and immune homeostasis which can lead to disease (Ubeda & Pamer, 2012). On the other hand, antibiotics can also positively modulate chronic disease conditions such as diabetes and obesity (Kootte et al, 2012). To what extent microbiota mediate the effects of antibiotics on metabolic disease remains an open question. One possibility is that exposure to antibiotics alters the structure and/or function of the microbiota in a way that promotes metabolic health. If fact, it has been shown that brief exposure, *e.g*. to kanamycin, chloramphenicol or tetracycline can alter the physiology and gene expression (stress response, proton motive force, antibiotic resistance and phage induction genes) of the microbiota in humans (Maurice et al, 2013).

**Bridge the Gap**

**The Gap**

As life-expectancy rises across the globe, the biological process of aging is an ever more predominant risk factor for the burden of disease, including metabolic, cardiovascular and neurodegenerative disease and cancer. Recent reports have identified a link between alterations to gastrointestinal tract microbiota and nutrition-related syndromes such as obesity and type-2 diabetes. However, many fundamental questions regarding the role of microbiota in disease aetiology remain unanswered. Are changes in microbiota the cause or the consequence of pathology? How does aging affect interactions between host and microbe in the context of disease? Is aging itself affected by alterations in these interactions? How may such complex interactions be investigated? Can interventions in host–microbe interactions provide protection against aging and the diseases integral to it? What is needed to answer many of these issues are model systems that can be used to clearly identify causative mechanisms and to directly test therapeutic interventions.

**The Bridge**

Here we argue that simple, defined host–microbiota model systems, such as *C. elegans* + *Escherichia coli*, can provide convenient, tractable and relevant additions to mammalian models (*e.g*. rodents) with which address these questions. Studies using lower animal models have a strong track record of elucidating the mechanisms of aging, metabolism and immunity. This Bridge the Gap article discusses the use of *C. elegans* to understand host–microbiota interactions, with emphasis on a neglected aspect of the biology of this intensively studied organism: its constitution as a nematode–microbe holobiont (or *worm-bug*).

**Figure 3:**
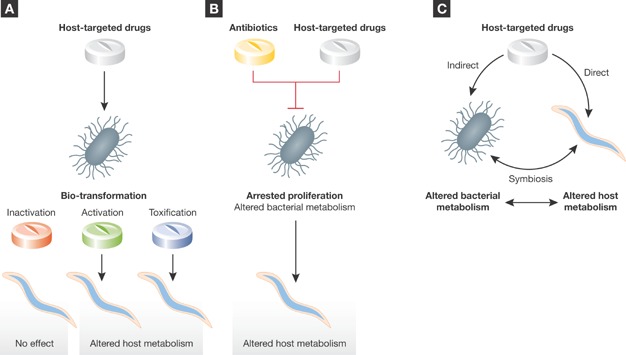
How to test host-targeted drugs in worms Bacterial roles in drug action on host physiology. Biotransformation (activation, inactivation, toxification) by different bacterial strains can alter the efficacy of a host-targeted drug with consequences to host physiology.Impact of host-targeted drugs and antibiotics on bacterial metabolism. Antibiotic and host-targeted drug action in bacterial proliferation and/or metabolism influences host physiology either through reduced pathogenesis or the alteration of bacterially derived metabolites.Host-targeted drugs can alter the physiology of the worm-bug. Effects of drugs on microbe and/or host can positively or negatively modulate the symbiotic relationship established between host and bacteria. For example, metformin action on *E. coli* can extend worm lifespan, but acting directly on worms metformin shortens lifespan (Cabreiro et al, 2013).

*C. elegans* is a convenient model for investigating the role of microbiota as a mediator of effects on drugs on the host. Drugs may affect the worm by altering nutritional, commensal or pathogenic properties of their partner microbes (Fig 3). Indeed, treatment of the worm-bug with the antibiotics ampicillin, kanamycin, sulfamethoxasole or trimethoprim increases lifespan (Cabreiro et al, 2013; Garigan et al, 2002; Virk et al, 2012).

Of particular interest is the role of microbiota in mediating effects on drugs targeted at metabolic disease. The composition of bacterial species in the gut can influence the course of diabetes and its treatment (Cani & Delzenne, 2009). Metformin is an anti-hyperglycaemic drug widely used to treat type-2 diabetes. In mammals, the action of metformin is partly mediated by AMPK activation, which results in down-regulation of mTOR and the IGF-1/AKT pathways to reduce energy-consuming processes (Pierotti et al, 2012). However, the benefits of metformin therapy go far beyond its prescribed usage, decreasing the risk of cancer (Dowling et al, 2011) and increasing lifespan in rodents (Anisimov et al, 2008). How exactly metformin exerts its beneficial effects under these conditions remains to be determined.

One possibility is that the drug alters mammalian physiology by changing the composition and/or metabolism of enteric microbes. Results of a number of studies support this interpretation. For example, metformin can alter gut microbial communities in rats (Pyra et al, 2012), although this study did not test the effect on host physiology of such alterations. In human subjects, 850 mg doses of metformin can generate concentrations of the drug >20 mM within the lumen of the intestine (Proctor et al, 2008). In fact, jejunal metformin concentration can reach a level that is 30–300 higher than that in the blood (Bailey et al, 2008). Such high concentrations of drug in the gut may well be responsible for the gastrointestinal side-effects (*e.g*. bloating and diarrhoea) that are a frequent and unfortunate consequence of metformin usage in humans (Bytzer et al, 2001).

In *C. elegans*, metformin slows down the aging process, an effect that is mediated by its effects on microbial metabolism (Cabreiro et al, 2013; Onken & Driscoll, 2010). Using the worm-bug we have found that metformin exerts DR effects on host lifespan by altering bacterial folate and methionine metabolism (Cabreiro et al, 2013). Notably, metformin can also cause folate deficiency in humans (Sahin et al, 2007). The case of metformin illustrates how important it is to consider the effects of drugs not only on the worm but also on the worm-bug. Metformin only shortens lifespan in the worm, but in the worm-bug it can increase or decrease lifespan depending on the strain of bacteria present. It is possible that, in addition to metformin, other drugs also alter bacterial metabolism to contribute to their therapeutic efficacy and/or possibly explain additional side effects.

In conclusion, recent insights into mammalian microbiota have directed attention of worm biologists to the relationship between *C. elegans* and its co-cultured microbes. This has brought into focus the multifaceted relationship between worm and microbe, and supports the view that lab cultured *C. elegans* is one component of a two organism holobiont. This dual organism, or worm-bug, is an excellent model with which to understand the role of microbiota in health and aging, DR and drug action.

## For more information

Human Microbiome Project

http://commonfund.nih.gov/hmp/

Kyoto Encyclopedia of Genes and Genomes (KEGG)

http://www.genome.jp/kegg/

Metagenomics of the Human Intestinal Tract

http://www.metahit.eu/

uBiome - Sequencing Your Microbiome

http://www.indiegogo.com/projects/ubiome-sequencing-your-microbiome
